# A cross-sectional study on the growth and development of preterm infants during the first year of life in Tibet’s Lhasa Region

**DOI:** 10.1097/MD.0000000000048138

**Published:** 2026-03-27

**Authors:** Qingqing Luo, Yangzong Suolang, Chenchen Bu, Ying Deng

**Affiliations:** a Department of Pediatrics, Women and Children’s Hospital of Tibet Autonomous Region, Lhasa, China; b Xizang Region Child Development Clinical Medical Research Center, Lhasa, China; c Department of Pediatrics, West China Second University Hospital, Sichuan University, Chengdu, China; d Key Laboratory of Birth Defects and Related Disease of Women and Children, Ministry of Education, Sichuan University, Chengdu, China.

**Keywords:** anemia, development, growth, high altitudes, preterm infants

## Abstract

Limited data exist on early growth and hematological parameters of preterm infants in high-altitude regions. This study was aimed to examine the growth and neurodevelopmental outcomes and the incidence of anemia and vitamin D deficiency (VDD) during the first year of life in preterm infants residing in Tibet’s Lhasa Region. This cross‑sectional study enrolled 228 preterm infants (including 98 small‑for‑gestational‑age [SGA] and 38 very preterm [VP] infants). At each follow‑up visit, 151, 117, 125, and 98 infants completed assessments at corrected ages (CA) of 1, 3, 6, and 12 months, respectively. Anthropometric measurements measured by trained health professionals at CA of 1, 3, 6, and 12 months. Neurodevelopment was assessed using the Infant Neurological Motor Assessment (20-item version, N20) at CA of 1, 3, and 6 months, the Alberta Infant Motor Scale at CA of 3 and 6 months, and the Developmental Screening Test at CA of 12 months. Venous blood samples were collected at CA of 6 and 12 months to determine hemoglobin (Hb, g/L) and serum 25-hydroxyvitamin D (ng/mL) levels. Anemia was defined as Hb < 137 g/L, and VDD was defined as serum 25-hydroxyvitamin D < 20 ng/mL. Preterm SGA infants showed significantly lower Z-scores for height, weight, and head circumference, with higher rates of underweight, stunting, and microcephaly. Similarly, VP infants displayed analogous patterns of compromised physical growth to preterm SGA infants. Furthermore, the SGA group exhibited a higher prevalence of development delay. Both SGA and VP birth were independently identified as significant risk factors for developmental delay. Anemia prevalence was 62.5% (35/56) at 6 months and 48.6% (35/72) at 12 months, while VDD rates decreased from 5% (2/40) to 3.4% (2/59) over the same period. In this high-altitude study, preterm infants as a group exhibited suboptimal growth indicators, a high prevalence of developmental delay, and a significant burden of anemia during their first year. These challenges were most severe in those born SGA or VP. Infants who were neither SGA nor VP showed better growth and neurodevelopmental outcomes. These findings advocate for enhanced surveillance and tailored interventions for all preterm infants, particularly high-risk subgroups, in high-altitude settings.

## 1. Introduction

The global preterm birth rate stands at 9.9%, while the incidence in China is 6.1%.^[1]^ Advances in perinatal care have markedly improved the survival rates of preterm infants; however, postnatal growth and developmental challenges remain critical concerns. VP (gestational age (GA) < 32 weeks) and SGA (birth weight/length < 10th percentile for GA) infants represent high-risk subgroups due to their distinct growth trajectories, which are strongly associated with long-term cardiometabolic and neurodevelopmental outcomes.^[2-4]^ Evidence indicates that SGA is associated with higher risks of growth retardation, neuropsychological impairment, obesity, insulin resistance, and metabolic syndrome in adulthood, significantly affecting long-term quality of life.^[5]^ Although most SGA infants achieve catch-up growth by 2 to 4 years of age, approximately 10% to 15% fail to do so. Failure to catch-up in height by 2 years of age increases the risk of short stature in adulthood by 5- to 7-fold.^[6]^ A study by Kong et al indicated that preterm infants with SGA have a significantly higher risk of specific developmental disorders (hazard ratio, 7.55 [95% CI, 6.61–8.62]).^[7]^

Under physiological conditions, infant growth follows a predetermined trajectory regulated by genetic, nutritional, and environmental factors. If pathological factors such as malnutrition or illness interfere, growth may falter, causing deviation from the normal trajectory. Once the constraining factors are removed, accelerated growth often occurs, allowing the child to return to the original growth channel – a process known as catch-up growth. While catch-up growth is common in preterm infants, particularly between 0 and 6 months of corrected age, both VP and SGA infants often show incomplete catch-up despite initial progress.^[3,4]^ Notably, excessively rapid catch-up growth may elevate the risk of metabolic syndrome in adulthood.^[8]^ VP and SGA infants are also more prone to insulin resistance and central obesity later in life.^[9]^ Thus, early and appropriate nutritional management is crucial for long-term health.

High-altitude environments have been consistently associated with growth deficits in children. Higher prevalences of underweight and stunting in high-altitude regions have been frequently reported.^[10]^ A study by Ma et al revealed that children aged 6 to 17 years living in Tibet had significantly lower height and weight compared to national reference standards.^[11]^ Stunting (12.3%) and underweight (9.2%) remain prevalent in this region.^[12]^ However, most existing studies have focused on school-age children, and data on early growth indicators in preterm infants in Tibet are limited.

Preterm infants are at increased risk of anemia due to reduced iron stores and high nutritional demands during rapid postnatal growth. The Qinghai–Tibet Plateau, with a mean elevation exceeding 3000 meters, presents a unique hypobaric-hypoxic environment that may disrupt iron homeostasis and reduce the efficacy of conventional iron supplementation regimens designed for sea-level populations. While oral iron supplementation at 2 mg/kg/d has proven effective in reducing anemia in low-altitude Chinese populations,^[13]^ its effectiveness in high-altitude settings remains unverified, underscoring the need for altitude-specific clinical evaluation.

This study aimed to assess growth, neurodevelopment, anemia, and VDD in preterm infants residing in Lhasa, Tibet. It focused on characterizing the high-risk SGA and VP subgroups within this high-altitude population, ultimately to generate evidence for more context-appropriate health monitoring and intervention strategies.

## 2. Materials and methods

### 2.1. Description of the infants

This cross-sectional study was conducted in the Department of Child Health at the Women and Children’s Hospital of Tibet Autonomous Region, located in Lhasa, Tibet, between January and December 2024. Preterm infants meeting the following criteria were enrolled: GA < 37 weeks; absence of major congenital malformations or genetic syndromes (e.g., Down syndrome). SGA was defined as birthweight (BW) or birthlength (BL) below the 10th percentile for GA according to the INTERGROWTH-21st standards (available at: https://intergrowth21.ndog.ox.ac.uk/). Infants with GA < 32 weeks were classified as VP infants.

### 2.2. Data collection

Neonatal and clinical data were retrospectively extracted from electronic medical records. Variables included GA, BW, BL, delivery mode (vaginal or cesarean), infant sex, and multiple birth status. Perinatal and postnatal complications were recorded, including bronchopulmonary dysplasia (BPD), intraventricular hemorrhage (IVH; grades I–IV), severe pneumonia (requiring mechanical ventilation), surgical intestinal disorders (e.g., necrotizing enterocolitis), neonatal respiratory distress syndrome, hypothyroidism (TSH > 10 mIU/L), and perinatal asphyxia. Maternal hypertensive disorders during pregnancy were also documented.

Post-discharge anthropometric measurements (weight, length, and HC) were obtained from records at (corrected age) CA of 1, 3, 6, and 12 months. CA was calculated by subtracting the weeks of prematurity from the chronological age to adjust for the expected 40-week gestation period, according to World Health Organization guidelines. Growth parameters were converted to standard deviation scores using the 2005 Chinese National Growth Charts.^[14]^ Underweight was defined as a weight-for-age Z-score (WAZ) less than -2 standard deviations (SD); stunting was defined as a length-for-age Z-score (LAZ) < −2 SD; and microcephaly was defined as a head circumference-for-age Z-score (HCZ) < −2 SD. BPD was defined as the requirement for supplemental oxygen for at least 28 days in infants born at <32 weeks of gestation. IVH was graded according to the Papile classification system (grade I–IV) based on cranial ultrasound findings.

This study utilized a cross-sectional design at each follow-up time point (CA 1, 3, 6, and 12 months). Data were retrospectively collected from scheduled clinical visits without a predefined longitudinal follow-up protocol.

### 2.3. Nutritional practices

All preterm infants received standardized iron prophylaxis (2 mg/kg/day) combined withvitamin D supplementation (800 IU/d until 3 months of CA, reduced to 400 IU/d thereafter). Feeding protocols were stratified by risk category:

1. Low-risk infants (BW > 2000 g, GA > 34 weeks): Exclusive breastfeeding or standard formula feeding was encouraged.
2. Moderate-risk infants (BW 1500–2000 g, GA 32–34 weeks): Breastfeeding was supplemented with fortified human milk or post-discharge formula until 3 months CA.
3. High-risk infants (BW < 1500 g, GA < 32 weeks): Breastfeeding was supplemented with fortified human milk or post-discharge formula until 6 months CA. Complementary feeding was initiated between 4 and 6 months CA, guided by individualized growth and neurodevelopmental assessments.^[13]^ This represents the standardized recommended practice in our country.

### 2.4. Evaluation tools

#### 2.4.1. N20

The Infant Neurological Motor Assessment (20-item version, N20), a validated tool for evaluating neurological development and motor function in infants,^[15]^ consists of 20 standardized tasks and observational items assessing neurological integrity across 4 domains: primitive reflexes, muscle tone (hypotonia, hypertonia), postural control, and behavioral responsiveness to stimuli. Clinicians routinely apply the N20 in high-risk populations (e.g., preterm infants, neonates with perinatal complications) to detect early signs of neurological abnormalities or developmental delays. Findings inform individualized early intervention plans and longitudinal neurodevelopmental surveillance to optimize outcomes. Failure to pass any single item indicates an abnormal neurological sign, necessitating further diagnostic evaluation. The N20 was administered to all infants at CA of 1, 3, and 6 months.

#### 2.4.2. Alberta Infant Motor Scale

AIMS is a standardized observational tool for assessing motor development in infants from birth to 18 months of age, validated for both term and preterm populations.^[16]^

Clinicians and researchers widely utilize the AIMS to longitudinally monitor motor skill acquisition and detect early signs of motor delay in high-risk infants. The scale evaluates motor performance across 4 positional domains: prone, supine, sitting, and standing, with additional scoring for movement quality (fluidity, symmetry) and maturational milestones. Raw scores are converted to percentile ranks using population-based normative data, enabling classification of an infant’s motor development relative to age-matched peers. Motor development is classified as follows – Atypical/Abnormal: percentile rank < 10 (indicating significant delay compared to chronological age norms). Motor development was assessed using the AIMS at CA of 3 and 6 months.

#### 2.4.3. Developmental screening test

The DST is a standardized tool designed to assess child development across 3 domains – motor, cognitive, and social-adaptive skills – in children aged 0 to 72 months.^[17]^ A DQ score < 85 indicates potential psychomotor delay, while scores ≥ 85 are classified as within the normal developmental range. In this study, the DST was administered at 12 months of CA to evaluate comprehensive developmental outcomes in preterm infants.

The N20, AIMS, and DST were administered by trained pediatricians and nurses within the Department of Child Health, all of whom had received standardized training on these tools.

#### 2.4.4. Hematological indicators

Venous blood samples (2.0ml) were collected from the infants at CA of 6 and 12 months. Samples were analyzed using a Sysmex XT-1800i automated hematology analyzer (Kobe, Japan) to quantify the following parameters: hemoglobin (Hb) concentration, mean corpuscular volume, hematocrit (HCT), mean corpuscular hemoglobin, and mean corpuscular hemoglobin concentration. Anemia was defined as Hb levels < 137 g/L at both 6 and 12 months.^[18]^ Serum 25-hydroxyvitamin D levels were measured using achemiluminescence immunoassay on a Savant-100 autoanalyzer (Beijing Savant Biotechnology Co., Ltd., China). VDD was defined as serum 25-hydroxyvitamin D levels < 20 ng/mL.^[19]^

#### 2.4.5. Statistical analysis

The data analysis followed a predefined plan to address 2 main objectives. First, to compare subgroups predefined (SGA vs non-SGA; VP vs non-VP) at each time point, continuous variables were compared using Student *t* test or Mann–Whitney *U* test, and categorical variables using the Chi-square or Fisher’s exact test, as appropriate. Second, to identify independent risk factors for developmental delay (DQ < 85) at 12 months, a multivariate binary logistic regression (Forward: LR method) was performed. Variables with a *P*-value < 0.1 in univariate analyses were considered for inclusion in the multivariate model. A 2-tailed *P*-value < .05 was considered statistically significant for all tests.

## 3. Results

**Figure 1.**
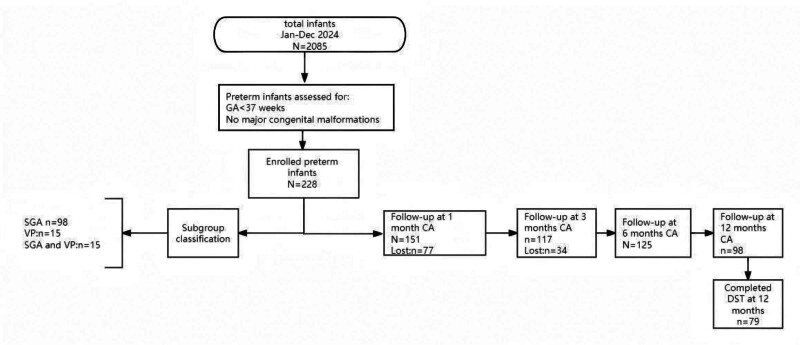
Participant flow diagram. Range reflects variable availability of anthropometric (weight, length, head circumference) data at each time point and DST at 12 months. CA = corrected age, DST = Developmental Screening Test, SGA = small for gestational age; VP = very preterm.

During the study period, there were 2085 infants, and a total of 228 preterm (10.9%) infants were enrolled, including 98 (43.0%) classified as SGA and 38 (16.7%) as VP infants. At corrected ages of 1, 3, 6, and 12 months, 151, 117, 125, and 98 preterm infants participated in the study assessments, respectively (Fig. 1). Baseline characteristics and clinical outcomes are summarized in Tables 1 and 2, respectively. At CA of 1, 3, 6, and 12 months, underweight prevalence was 13.2, 19.7, 24.0, and 15.3%, respectively. Stunting rates were 13.3, 13.7, 15.2, and 13.3%,while microcephaly incidence increased progressively from 4. 1% at 1 month to 10.3% at 12 months.

**Table 1:** Baseline neonatal and demographic characteristics of the study cohort.

Variables	% or mean ± SD
Male	50.8 (116/228)
VP infants	16.7 (38/228)
Cesarean	70.2 (160/228)
Twins	9.6 (22/228)
SGA	43 (98/221)
GA (wk)	34.3 ± 2.4
BW (g)	2009 ± 627
BL (cm)	43.3 ± 5.4
Z-score for BW	−0.68 ± 1.16
Z-score for BL	−0.56 ± 1.67
NRDS	2.2 (5)
IVH	0.9 (2)
Pneumonia	2.2 (5)
Asphyxia	3.5 (8)
Hypertensive disorders in pregnancy	2.2 (5)
Hypothyroidism	1.8 (4)
BPD	0.4 (1)
Surgical intervention	0.4 (1)

BL = birth length, BPD = bronchopulmonary dysplasia, BW = birth weight, GA = gestational age, IVH = intraventricular hemorrhage, NRDS = Neonatal respiratory distress syndrome, SD = standard deviations, SGA = small for gestational age, VP = very preterm.

**Table 2:** The prevalence of underweight, stunting, microcephaly and developmental delay was assessed at various corrected ages (%, n).

	Underweight	Stunting	Microcephaly	N20	Alberta	DST
1 mo	13.2 (20/151)	13.3 (20/150)	4.1 (6/147)	71.1 (86/121)	–	–
3 mo	19.7 (23/117)	13.7 (16/117)	6.3 (7/111)	51.8 (43/83)	25.9 (15/58)	–
6 mo	24 (30/125)	15.2 (19/125)	9.6 (12/123)	29.3 (17/58)	39 (23/59)	–
12 mo	15.3 (15/98)	13.3 (13/98)	10.3 (10/97)	–	–	45.6 (36/79)

Alberta = Alberta infant motor scale, DST = Developmental Screening Test, mo = months, N20 = Infant Neurological Motor Assessment 20 Items.

Neurodevelopmental assessments revealed declining abnormal N20 findings: 71. 1% at 1 month, 51.8% at 3 months, and 29.3% at 6 months of CA. AIMS-identified motor delays affected 25.9% (15/58) of infants at 3 months and 39.0% (23/59) at 6 months. By 12 months CA, the DST identified developmental delay in 45.6% (36/79) of infants. Subgroup analyses demonstrated significantly higher developmental delay rates at 12 months in SGA versus non-SGA infants (62.9% vs 31.8%; *P* < .05). Although VP infants showed higher delay prevalence than non-VP infants (69.2% vs 40.9%), this difference was not statistically significant (*P* = .061). Notably, at 6 months CA, VP infants had significantly higher motor delay prevalence than moderate-to-late preterm infants (75.0% vs 33.3%; *P* = .025; Table 3).

**Table 3:** Comparison of growth retardation and developmental delay assessments between the groups (%, n).

	Non-SGA	SGA	*P*	Non-VP infants	VP infants	*P*
1 Mo						
Underweight	7.6 (6/79)	19.7 (14/71)	.029	11.2 (14/125)	23.1 (6/26)	.104
Stunting	5.1 (4/79)	22.9 (16/70)	.001	9.7 (12/124)	30.8 (8/26)	.004
Microcephaly	2.6 (2/77)	5.8 (4/69)	.001	1.7 (2/121)	15.4 (4/26)	.001
N20	64.4 (38/59)	78.7 (48/61)	.083	72.3 (73/101)	65 (13/20)	.512
3 mo						
Underweight	9 (6/67)	34 (17/50)	.001	14.9 (13/87)	33.3 (10/30)	.029
Stunting	6 (4/67)	24 (12/50)	.005	6.9 (6/87)	33.3 (10/30)	<.0001
Microcephaly	3.1 (2/64)	10.6 (5/47)	.108	3.6 (3/83)	14.3 (4/28)	.045
N20	59.2 (29/49)	41.2 (14/34)	.106	51.8 (29/56)	51.9 (14/27)	.995
Alberta (*P* < 10)	20 (6/30)	32.1 (9/28)	.291	25.5 (12/47)	27.3 (3/11)	.906
6 mo						
Underweight	12.3 (8/65)	36.7 (22/60)	.001	19.2 (20/104)	47.6 (10/21)	.005
Stunting	3.1 (2/65)	28.3 (17/60)	<.0001	12.5 (13/104)	28.6 (6/21)	.061
Microcephaly	3.1 (2/64)	16.9 (10/59)	.01	5.9 (6/102)	28.6 (6/21)	.001
N20	29.4 (10/34)	29.2 (7/24)	.984	26.5 (13/49)	44.4 (4/9)	.278
Alberta (*P* < 10)	27.6 (8/29)	50 (15/30)	.078	33.3 (17/51)	75 (6/8)	.025
12 mo						
Underweight	7 (4/57)	26.8 (11/41)	.007	11 (9/82)	37.5 (6/16)	.007
Stunting	3.5 (2/57)	26.8 (11/41)	.001	8.5 (7/82)	37.5 (6/16)	.002
Microcephaly	5.4 (3/56)	17.1 (7/41)	.061	4.9 (4/81)	37.5 (6/16)	<.0001
DQ < 85	31.8 (14/44)	62.9 (22/35)	.006	40.9 (27/66)	69.2 (9/13)	.061

Alberta = Alberta infant motor scale, DQ = developmental quotient, DST = Developmental Screening Test, Mo = months, N20 = Infant Neurological Motor Assessment 20 Items, SGA = small for gestational age, VP = very preterm.

Multivariate logistic regression confirmed both SGA status (OR = 4. 152; 95% CI = 1.558–11.063, *P* = .004) and very preterm birth (OR = 4. 137; 95% CI = 1.062–16. 114, *P* = .041) as independent risk factors for developmental delay.

### 3.1. A comparative analysis of growth status among groups

All anthropometric measurements at CA of 1, 3, 6, and 12 months were consistently lower in SGA infants compared to non-SGA infants (Table 4). Preterm SGA infants demonstrated significantly higher prevalence rates of underweight, stunting, and microcephaly at 1, 6, and 12 months CA (Table 4).

**Table 4:** The growth pattern between different groups (mean ± SD).

	Non-SGA	SGA	*P*	Non-VP infants	VP infants	*P*
1 mo						
WAZ	−0.31 (1.05)	−1.33 (0.92)	<.0001	−0.71 (1.08)	−1.09 (1.28)	.121
LAZ	−0.21 (0.91)	−1.29 (1.53)	<.0001	−0.6 (1.35)	−1.24 (1.22)	.029
HCZ	0.16 (1.04)	−0.64 (0.74)	<.0001	−0.11 (0.83)	−0.65 (1.5)	.005
3 mo						
WAZ	−0.78 (0.98)	−1.57 (0.77)	<.0001	−0.95 (0.9)	−1.61 (1.05)	.001
LAZ	−0.54 (1.06)	−1.37 (0.95)	<.0001	−0.68 (0.97)	−1.53 (1.2)	<.0001
HCZ	−0.53 (0.84)	−0.98 (0.78)	.005	−0.59 (0.77)	−1.11 (0.94)	.004
6 mo						
WAZ	−0.54 (1.18)	−1.73 (0.93)	<.0001	−0.95 (1.16)	−1.87 (1.26)	.001
LAZ	−0.18 (1.21)	−1.44 (1.03)	<.0001	−0.64 (1.25)	−1.51 (1.29)	.005
HCZ	−0.34 (0.86)	−1.01 (1.01)	<.0001	−0.49 (0.92)	−1.48 (0.93)	<.0001
12 mo						
WAZ	−0.57 (1.14)	−1.33 (0.92)	.001	−0.73 (1.02)	−1.69 (1.25)	.001
LAZ	−0.38 (1.31)	−1.1 (1.02)	.004	−0.51 (1.11)	−1.56 (1.54)	.002
HCZ	−0.28 (1.15)	−0.75 (1.08)	.044	−0.35 (1.07)	−1.13 (1.26)	.011

HCZ = head circumference-for-age Z-score, LAZ = length-for-age Z-score, SGA = small for geatational age, VP = very preterm, WAZ = weight-for-age Z-score.

In the VP infants group, WAZ, LAZ, and HCZ at 3, 6, and 12 months CA were significantly lower than in the non-VP infants group (Table 4). At 1 month CA, VP infants exhibited significantly lower Z-scores for length and HC compared to non-VP infants, with higher rates of stunting and microcephaly than moderate-to-late preterm infants. These disparities persisted at 3 and 12 months CA, with VP infants showing significantly higher prevalence of underweight, stunting, and microcephaly (Table 4).

### 3.2. The level of hematological indicators among preterm infants

Hematological parameters were assessed through venous blood sampling at CA of 6 and 12 months. Complete blood test data were available for 56 infants at 6 months and 72 infants at 12 months. Median Hb concentrations were 134 g/L (interquartile range [IQR]: 125–145 g/L) at 6 months and 137 g/L (IQR: 128–144 g/L) at 12 months. Similarly, median VD levels were 44.3 ng/mL (IQR: 31.8–52.0 ng/mL) and 44.8 ng/mL (IQR: 34.9–52.8 ng/mL) at 6 and 12 months of CA, respectively (Table 5). The prevalence of anemia was 62.5% (35/56) at 6 months, declining to 48.6% (35/72) at 12 months. VDD was detected in 5.0% (2/40) of infants at 6 months, with a slight reduction to 3.4% (2/59) at 12 months.

**Table 5:** Blood routine parameters and VD levels were analyzed across different CA groups (Median [IQR]).

	P25	Median	P75
6 mo			
Hb	125	134	145
HCT	37.1	39.9	43.7
MCV	78.5	81.9	86.7
MCH	26.2	27.2	28.8
MCHC	325	334	340
VD	31.8	44.3	52
12 mo			
Hb	128	137	144
HCT	38.6	40.8	43
MCV	76.8	78.7	81.1
MCH	25.7	26.6	27.3
MCHC	328	335	343
VD	34.9	44.8	52.8

CA = corrected age, Hb = hemoglobin, HCT = hematocrit, MCH = mean corpuscular hemoglobin, MCHC = mean corpuscular hemoglobin concentration, MCV = mean corpuscular volume, median (IQR) = median (P25, P75), VD = vitamin D.

## 4. Discussion

Tibet, located on the world’s highest plateau, encompasses extensive regions (86% of its total area) at altitudes exceeding 4000 meters. The environmental conditions of this region are defined by chronic hypoxia, low atmospheric pressure, and extreme cold. These unique geographical features have profoundly influenced both the cultural practices and social dynamics of local populations. Our study focused on Lhasa, the capital of Tibet, which lies at an average altitude of 3600 meters. As the cultural and demographic center of Tibet, Lhasa provides a critical setting for investigating the health impacts of high-altitude environment on vulnerable populations, particularly preterm infants. Our results demonstrate considerable disparities in growth and neurodevelopmental outcomes among preterm infants during their first year of life in this high-altitude setting. At 12 months of corrected age, the prevalence of underweight, stunting, and microcephaly in our study was 15.3, 13.3, and 10.3%, respectively. These figures are lower than those reported by Dang et al in 2004 among Tibetan children under 3 years of age, in which underweight affected 24.7% and stunting 39% of the subjects.^[20]^ This discrepancy suggests that improvements in nutritional support and healthcare over recent years may have contributed to a reduction in malnutrition rates. Nevertheless, the prevalence of malnutrition in high-altitude regions remains substantially higher than that in low-altitude areas. For instance, our previous single-center study of over 800 preterm infants in a low-altitude setting reported rates of underweight, stunting, and microcephaly of only 2.4, 1.1, and 0.9%, respectively, at 1 year of corrected age.^[21]^ High-altitude exerts an independent adverse effect on growth. Even under favorable socioeconomic conditions, children residing at altitudes above 1500 meters exhibit significantly lower height-for-age Z-scores. Each 500-meter increase in altitude is associated with an average decrease of 0. 163 in Z-scores, confirming high-altitude as an independent risk factor for malnutrition.^[22]^ Specifically, preterm infants classified as SGA or VP infants exhibited significantly lower Z-scores for weight, length, and HC compared to non-SGA and moderate-to-late preterm infants, respectively. These results underscore the higher risk of malnutrition in high-risk preterm subgroups, even when compared to populations in low-altitude settings. Consistent with previous research, our data align with studies demonstrating the persistent growth challenges faced by SGA and VP infants. For example, a cohort study in Chengdu identified SGA and very low birth weight (<1500 g) as independent risk factors for impaired growth during the first year of life in preterm infants.^[21]^ Similarly, Fuentefria et al reported that VP infants in Brazil exhibited significantly slower growth trajectories compared to full-term infants.^[4]^

Current evidence demonstrates that neonates in high-altitude regions face a 40% increased risk of low birth weight (<2500 g) compared to low-altitude counterparts, with birth weights averaging 100 to 120 g lower.^[19,20]^ In our cohort of 228 preterm infants, the incidence of SGA (43%) was more than double the maximum rate reported in low-altitude studies (20.9% in 834 preterm very low birth weight infants).^[21]^ This disparity highlights the urgent need for interventions to optimize intrauterine growth in high-altitude pregnancies. Altitudes ≥ 3500 meters have been shown to significantly impair childhood linear growth, as observed in both Tibetan and Andean populations.^[23-25]^ Notably, the risk of stunting increases 2- to 6-fold per 1000-meter elevation gain,^[26]^ likely due to hypoxia-induced metabolic dysregulation and nutrient malabsorption.^[23,27]^ While underweight and stunting typically worsen post-infancy,^[28]^ our findings indicate that these growth impairments are already prominent during the first year of life, necessitating early targeted interventions.

Despite standardized iron supplementation, anemia prevalence remained alarmingly high in our cohort, peaking at 62.5% at 6 months of CA and decreasing to 48.6% at 12 months. This aligns with Tibetan Shannan’s 57.84% anemia prevalence in 0 to 6-year-olds,^[29]^ but far exceeds rates observed in low-altitude regions (<10%).^[21]^ Current data on childhood anemia in high-altitude regions remain limited, particularly regarding preterm infants. A Peruvian study involving 20,28,701 children aged 6 to 59 months demonstrated that after adjusting the WHO threshold for altitude, anemia prevalence was ~36% below 1000 m and increased to ~66% above 4000 m.^[30]^ High-altitude residency (>4000 m) itself is an independent risk factor for anemia (OR = 1.88).^[29]^ Anemia in these regions is a risk factor for other conditions, including reduced serum ferritin, which is an independent risk factor for right heart failure in Tibet (OR = 5.85, 95% CI 1.59–21.59).^[31]^ Anemia also interacts with diseases such as pneumonia, increasing the risk of treatment failure by 4.07 times in high-altitude children.^[32]^ Although our study is limited by its sample size, it offers foundational data for establishing altitude-specific hematological reference ranges. These findings provide valuable insights for developing region-specific anemia reference standards^[33]^ and underscore the importance of iron supplementation, particularly for children in rural and pastoral areas.^[29,34]^ Paradoxically, despite Tibet’s historically high rickets prevalence (66%) linked to VDD,^[35]^ our cohort showed remarkably low VDD rates (5% at 6 months; 3.4% at 12 months), underscoring the success of systematic supplementation programs even in high-risk populations.

Neurodevelopmental research in high-altitude preterm infants remains scarce. Available evidence suggests that higher altitudes (especially above 2500 meters) are associated with an increased risk of neurodevelopmental issues. A cohort study of 2116 infants across 5 South American countries found that for every 100-meter increase in altitude, the risk of being classified as high-risk for neurodevelopmental problems rose by 2% (OR 1.02), with the risk further elevated at altitudes above 2500 meters (OR 1.35). This indicates that chronic hypoxia may interfere with early neurodevelopment.^[36]^ Our study observed the highest rate of abnormal neurodevelopmental scores at 1 month CA (71. 1%), declining to 29.3% by 6 months. A study using the Bayley Scales of Infant Development demonstrated that no significant differences in neurodevelopmental outcomes across altitude groups, suggesting the presence of adaptive protective mechanisms during infancy, such as increased hemoglobin concentration or cerebrovascular remodeling.^[37]^ However, motor delays assessed by the AIMS paradoxically increased from 25.9% at 3 months to 39.0% at 6 months, potentially due to environmental factors (e.g., limited motor stimulation in cold climates) or methodological constraints. Moreover, we observed an increase in the frequency of microcephaly with age, while at the same time noting a decrease in abnormal N20. This apparent contradiction may arise from differences in assessment tools and the nature of the measures. The N20 evaluates neurological signs that may improve with maturation, while microcephaly reflects a structural growth deficit that may become more apparent over time. Additionally, as a cross-sectional study, different infants may have been assessed at each time point, and those with more severe impairments might have been lost to follow-up. By 12 months, 45.6% of infants exhibited developmental delay, exceeding low-altitude benchmarks,^[38]^ and highlighting potential long-term consequences of hypoxic adaptation strategies. Preclinical models suggest hypoxia alters neural stem cell differentiation, suppressing astrogenesis and promoting oligodendrocyte overproduction, leading to myelination defects.^[39]^ Although the univariate comparison showed a higher prevalence of developmental delay in VP infants (69.2%) compared to non-VP infants (40.9%), this difference did not reach the conventional threshold for statistical significance (*P* = .061). This borderline result may reflect limited statistical power due to the relatively small sample size in the VP subgroup. But both SGA and VP status independently predicted neurodevelopmental delays, consistent with studies linking SGA to hyperactivity,^[40]^ and VP to motor deficits (32–42%), cognitive impairment (26%), intellectual disability (19%) and language delays.^[41-44]^ Latent profile analysis revealed that approximately 15% of VP infants belong to a “high-risk group” characterized by cognitive, motor, and behavioral impairments at 2 years of age.^[45]^ Additionally, the risk of autism spectrum disorder is elevated in this population.^[46]^ These findings underscore the imperative for: enhanced surveillance for SGA/VP infants; early interventions targeting sensory-motor pathways during the critical neuroplasticity window (0–2 years); family education to promote developmental activities in high-altitude environments.^[41,47]^

### 4.1. Limitations and strengths

A key limitation of this study is its restriction to a single follow-up assessment time point, which precludes longitudinal analysis of growth patterns. A considerable number of infants missed 1 or more scheduled visits, resulting in a pattern of missing data that is unlikely to be “missing completely at random.” Relatedly, we did not perform sensitivity analyses to quantify how such missing data might influence the robustness of our primary conclusions, particularly the identified risk factors for developmental delay. While this study provides novel insights into Tibetan preterm infants at high-altitude, the cross-sectional nature of assessments at each time point precludes analysis of individual growth trajectories or causal inferences regarding altitude effects, and the modest sample size (n = 228) limits power for subgroup analyses (e.g., by GA strata or hematological adaptation phenotypes). Excluding infants with congenital anomalies may underestimate developmental delays, while single-center data from Lhasa restrict generalizability to other high-altitude populations. Moreover, substantial attrition throughout the follow-up period may have introduced selection bias, as infants with more severe health conditions or those residing in geographically remote regions were less likely to complete scheduled assessments. This potential attrition bias could compromise the external validity of our findings. To address these limitations, future research should prioritize large-scale, multi-center longitudinal studies incorporating robust participant retention strategies (tailored follow-up protocols, community-based engagement) and thorough sensitivity analyses to evaluate the potential impact of missing data on study outcomes. Such methodological refinements would strengthen the generalizability and reliability of the conclusions.

## 5. Conclusions

In conclusion, SGA and VP status are key independent risk factors for developmental delay in Tibetan preterm infants. These infants demonstrated suboptimal growth trajectories during their first postnatal year. Despite standardized iron supplementation, anemia prevalence remained elevated in high-altitude preterm infants, underscoring the urgent need for altitude-adjusted iron protocols. Conversely, systematic vitamin D supplementation effectively mitigated VDD. These findings emphasize the necessity of early nutritional surveillance, targeted iron supplementation strategies tailored to high-altitude physiology, and sustained vitamin D prophylaxis to optimize neurodevelopmental and growth outcomes in vulnerable populations residing at extreme elevations.

## Author contributions

**Conceptualization:** Yangzong Suolang.

**Data curation:** Yangzong Suolang.

**Formal analysis:** Qingqing Luo, Yangzong Suolang.

**Investigation:** Chenchen Bu.

**Methodology:** Qingqing Luo, Yangzong Suolang.

**Software:** Chenchen Bu.

**Writing – original draft:** Ying Deng, Qingqing Luo, Yangzong Suolang.

**Writing – review & editing:** Ying Deng.
